# Long non-coding RNA HCP5 functions as a sponge of miR-29b-3p and promotes cell growth and metastasis in hepatocellular carcinoma through upregulating DNMT3A

**DOI:** 10.18632/aging.203155

**Published:** 2021-06-18

**Authors:** Yongping Zhou, Kuan Li, Tu Dai, Hong Wang, Zhiyuan Hua, Wuyang Bian, Hao Wang, Fangming Chen, Xiaoming Ai

**Affiliations:** 1 Department of Hepatobiliary Surgery, Wuxi Second Hospital, Nanjing Medical University, Wuxi, Jiangsu, China; 2 Department of General Surgery, BenQ Medical Center, The Affiliated BenQ Hospital of Nanjing Medical University, Nanjing, Jiangsu, China; 3 Department of Imaging, Wuxi Second Hospital, Nanjing Medical University, Wuxi, Jiangsu, China; 4 Department of Hepatobiliary Surgery, Kunshan Hospital of Traditional Chinese Medicine, Kunshan, Jiangsu, China

**Keywords:** lncRNA HCP5, hepatocellular carcinoma, miR-29b-3p, DNMT3A, metastasis

## Abstract

Multiple studies have revealed that long non-coding RNA (lncRNAs) served as regulatory factors in modulating tumorigenesis of hepatocellular carcinoma (HCC). In the present study, we demonstrated that lncRNA HCP5 was overexpressed in HCC tissues and cell lines, and these findings were obvious even in metastatic and recurrent cases. Knockdown of HCP5 significantly alleviated cell growth, metastasis, and invasion both *in vitro* and *in vivo* through promoting apoptosis and by inactivating the epithelial-mesenchymal transition (EMT) progress. Moreover, miR-29b-3p has been identified as a negatively regulatory target gene of HCP5, and served as a tumor suppressor of HCC to prevent cell proliferation, migration, and invasion. Subsequently, DNMT3A was identified as a downstream regulatory factor of miR-29b-3p, and acted as a participated element of HCC progression by activating AKT phosphorylation. Taken together, our study elucidated for the first time that HCP5 plays a crucial role in HCC via the HCP5/miR-29b-3p/DNMT3A/AKT axis and our findings demonstrated a novel diagnostic and therapeutic strategy with potentiality to treat HCC.

## INTRODUCTION

Liver cancer is a common digestive malignant cancer worldwide, and hepatocellular carcinoma (HCC) is the predominant histological type of liver cancer. HCC has been ranked as the third leading cause of cancer mortality with a high incidence rate [1]. It has been reported that annually, roughly seven hundred thousand people die of HCC worldwide [2]. Although multiple treatment methods have been employed in curing HCC, its poor prognosis and high degree of malignancy still limit improving the lifespan of HCC patients [3]. Additionally, due to the lack of early symptoms and specific biomarkers, a majority of HCC patients is already in the advanced phase or have developed metastases when being diagnosed [4]. Generally, migration to peripheral or distant tissues is recognized as the pivotal factor for recurrence of HCC [5]. Because of its aggressiveness and easy recurrence, traditional treatment methods, such as surgical resection, liver transplantation, radiotherapy, and chemotherapy, lack efficient treatment effects. Therefore, identifying biomarkers for early diagnosis and therapy strategies has become the research focus in the HCC field.

Recently, in many studies, it was discovered that there are some pathological correlations between long non-coding RNA (lncRNAs) and various malignant carcinomas, including gastric cancer, breast cancer, colorectal cancer, and non-small cell lung cancer [6–10], which make lncRNAs a hotspot of cancer therapy fields. Accumulating evidence has shown that some lncRNAs function as a sponge of specific microRNAs (miRNAs) and therefore mediate the tumorigenesis process of HCC. Research performed by He et al. illustrated that lncRNA maternally expressed gene-3 (MEG-3) prevented cell proliferation, and the metastatic and invasive capacity via negatively regulating miR-664 [11]. Moreover, Li et al. demonstrated that metastasis-associated lung adenocarcinoma transcript 1 (MALAT-1) also functioned as a molecular sponge of miR-146b-5p and therefore promoted cancer cell growth and invasion of HCC [12].

Human histocompatibility leukocyte antigen (HLA) complex P5 (HCP5) has been recognized as a key factor in cells of the immune system with potential regulatory effects in autoimmunity [13]. So far, many studies have proposed a pathological association between HCP5 and various cancers. It has been suggested that HCP5 could promote the progression of colorectal cancer by the miR-299-3p/PFN1/AKT axis, thereby exacerbating triple negative breast cancer by acting as a ceRNA to modulate BIRC3 and down-regulate miR-219a-5p. This facilitates the development of cervical cancer by regulating MACC1 via suppression of microRNA-15a and accelerate follicular thyroid carcinoma progression via miRNA sponges [14–17]. Additionally, knockdown of HCP5 could exert tumor-preventing effects by up-regulating miR-128-3p in anaplastic thyroid cancer [18]. Although HCP5 has been reported as a susceptibility locus for HCV-related HCC in meta-analyses by Christian et al. [19], the exact function of HCP5 in HCC remains to be elucidated.

In recent decades, miRNAs have been revealed as tumor regulatory factors, which participate in many processes, including cell cycle, apoptosis, and epithelial-stromal transformation [20, 21]. Jinmal et al. indicated that the expression of abbreviated miRNAs is pathologically correlated to HCC by a hepatitis infection, cirrhosis, and patient survival [22], which indirectly suggested the participation of miRNAs in HCC. Recently, miR-29b-3p was proven as the tumor promoting factor of bladder cancer via suppressing DNA methyltransferase 3A (DNMT3A) [23]. Although the function of miR-29b-3p was observed in many types of cancers, including bladder cancer, colorectal cancer, and pancreatic cancer [24, 25], there is the specific function of miR-29b-3p in HCC is still unknown, which encouraged us to further investigate the underlying signal pathway in HCC.

DNMT3A is a member of the DNA methyltransferase (DNMTs) family, and is known as a crucial factor in the tumor epigenetic mechanism that functions in de novo methylation [26]. It has previously been reported that DNTM3A could methylate the promoter region of the PTEN gene, thereby resulting in inhibition of transcription of the PTEN gene and decreased expression of the PTEN protein, finally elevating the phosphorylation level of AKT [27]. Data on the participation of DNMT3A in various types of carcinomas are now widely accepted and DNMT3A has been reported as a factor that is closely related to lung cancer [28], colorectal cancer [29], ovarian cancer [30], and liver cancer [31]. However, whether there is pathological association between lncRNA the HCP5/miR-29b-3p/DNMT3A axis and HCC is still not known and needs further investigation. This study was designed to give identify the regulatory function of the HCP5/miR-29b-3p/DNMT3A axis in HCC.

## RESULTS

### LncRNA HCP5 was highly expressed in HCC tissues and cell lines and closely correlated with HCC progression

**Figure 1:**
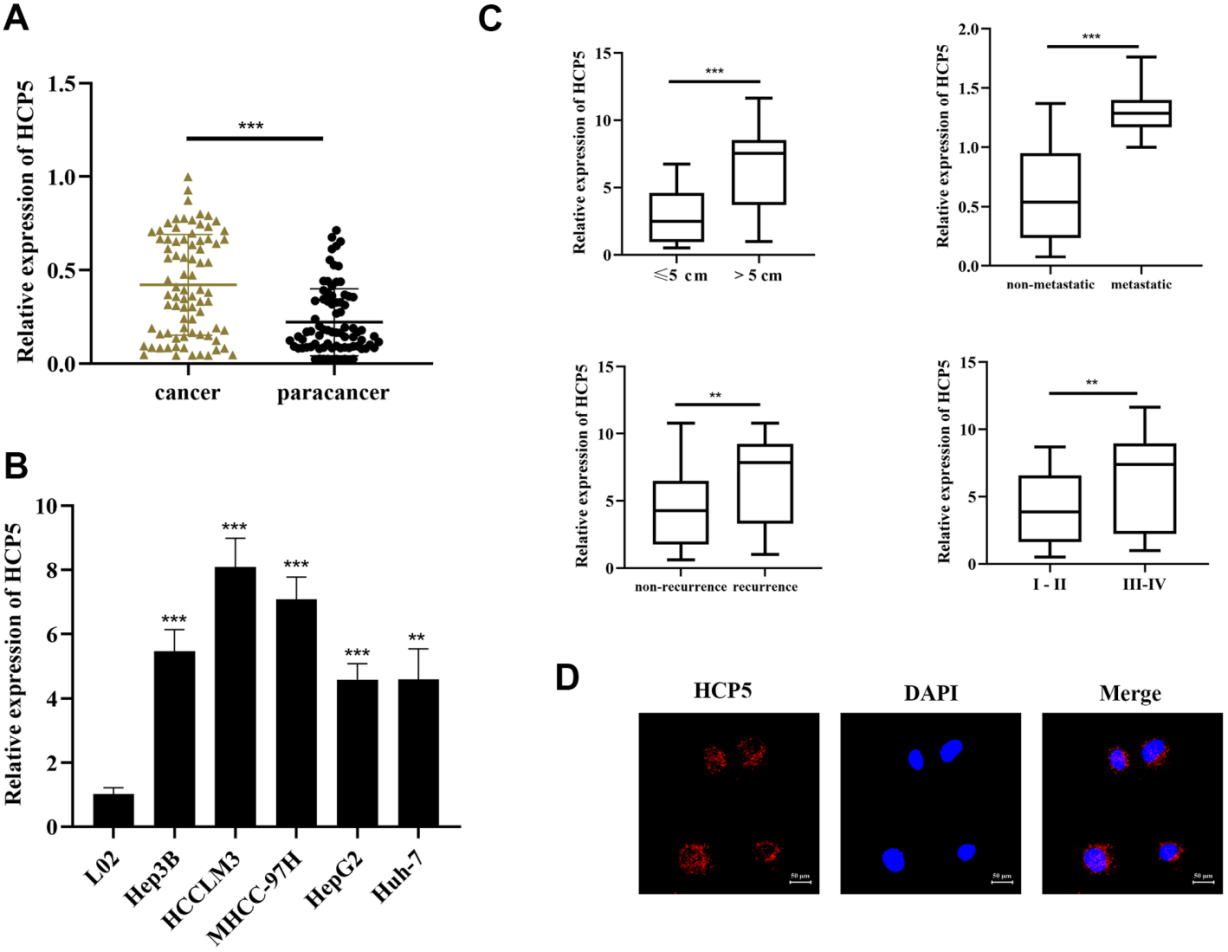
**LncRNA HCP5 is overexpressed in HCC tissues and cell lines.** (**A**) The expression level of HCP5 in HCC tissues is significantly higher compared to that in paracancerous tissues (***p<0.001, GAPDH used served as the internal control). (**B**) HCP5 is overexpressed in HCC cell lines (***p<0.001, **p<0.01), and the expression level is low in normal hepatocytes. (**C**) The expression of HCP5 in tumors with a size >5 cm, migrated HCC tissues, recurrent cancer tissues, and high histological grade tumor tissues was significantly higher (**p<0.01, ***p<0.001). (**D**) FISH assay indicating that HCP5 was predominantly localized in the cytoplasm of Hep3B cell lines.

To examine the HCP5 expression in HCC-related tissues and cell lines, qRT-PCR was conducted to determine 80 pairs of stochastically extracted HCC tissues and adjacent normal tissues. In the present study, we showed that HCP5 was overexpressed in HCC tissues versus adjacent normal tissues (p<0.001, Figure 1A, GAPDH served as the internal control). Simultaneously, HCP5 was expressed at a level in HCC cell lines (Hep3B, HCCLM3, HepG2, Huh7, and MHCC-97H) when compared with normal LO2 hepatocytes (***p<0.001, **p<0.01, respectively, Figure 1B). Thus, these findings collectively suggested that overexpression of lncRNA HCP5 might be associated with HCC.

Next, we further examined the association between clinical parameters and HCP5 expression. As shown in Table 1, HCP5 was significantly higher expressed in large tumors, metastasis, high histological grade tissues, and recurrence (**p<0.01, ***p< 0.001, Figure 1C). Furthermore, the result of FISH analysis demonstrated that HCP5 was predominantly localized in the cytoplasm of Hep3B cells (Figure 1D). Together, these results showed that HCP5 was closely associated with a higher-grade malignance and HCP5 likely plays an important role in regulating the progression of HCC.

**Table 1:** Association analysis of lncHCP5 expression and the clinicopathological features in 80 HCC patients.

**Characteristics**	**Case number**	**lncHCP5**		***P*-value**
		**Low** **(n=40)**	**High** **(n=40)**	
Number	80	40	40	
Ages (years)				0.4957
< 65 years	47	25	22	
≥ 65 years	33	15	18	
Gender				0.1990
Male	61	33	28	
Female	19	7	12	
AFP, μg/L				0.4123
<400 ng/ml	17	7	10	
≥ 400 ng/ml	63	33	30	
Cirrhosis				0.5762
Absent	16	9	7	
Present	64	31	33	
Tumor size				0.0008
≤ 5 cm	32	23	9	
> 5 cm	48	17	33	
Tumor number				0.1305
Single	46	27	22	
Multiple	34	13	21	
Vascular invasion				0.0013
Absent	18	3	15	
Present	62	37	25	
TNM stage				0.0116
I - II	49	30	19	
III-IV	31	10	21	
Edmondson				0.0005
I - II	51	33	18	
III + IV	29	7	22	
Capsular				
Present	53	25	28	0.4781
Absent	27	15	12	

^a^Two-sided chi-squared test.

The median expression level was used as the cut-off.

### HCP5 promotes cell growth, metastasis, and invasion in HCC *in vitro* and *in vivo* via inhibiting cell apoptosis and enhancing EMT

**Figure 2:**
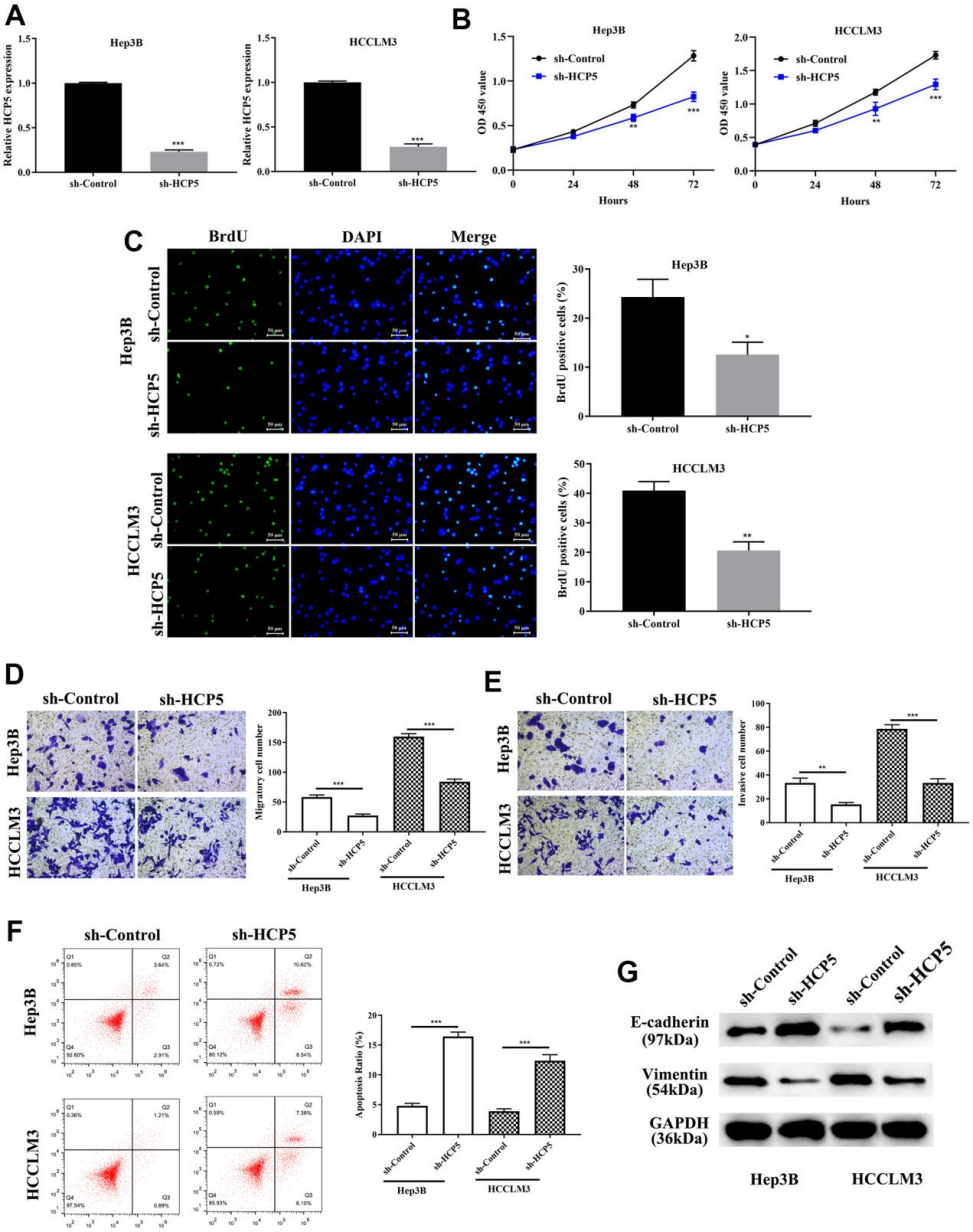
**LncRNA HCP5 augments cell growth, metastasis, invasion, the epithelial-mesenchymal transition (EMT) process and prohibits hepatocellular carcinoma (HCC) cell apoptosis.** (**A**) Construction of HCP5 knockdown in Hep3B and HCCLM3 cell lines was successful using sh-HCP5 (***p<0.001, GAPDH served as the internal control). (**B**, **C**) Compared to the sh-control, knockdown of HCP5 in HCCLM3 and Hep3B cells prohibited cell proliferation by the CCK-8 assay and BrdU assay (*p<0.05, **p<0.01, ***p<0.001). (**D**, **E**) Down-regulation of HCP5 decreased migration and invasion in HCCLM3 and Hep3B cell lines (***p<0.001, **p<0.01). (**F**) Cell apoptosis was promoted in HCP5 knockdown cells (***p<0.001). (**G**) Western blot showing that EMT-related protein E-cadherin was overexpressed in the HCP5 down-regulated group while the expression of vimentin was inhibited.

Investigation on the specific function of HCP5 in HCC was performed by transfecting sh-HCP5 into Hep3B and HCCLM3 cell lines to knockdown HCP5. As depicted in Figure 2A, HCP5 was significantly lower expressed in HCC cell lines (Hep3B and HCCLM3) that underwent sh-HCP5 transfection. Subsequently, the cell proliferative capacity was attenuated in Hep3B and HCCLM3 cell with HCP knockdown using the CCK-8 assay (**p<0.01, ***P<0.001, Figure 2B) and BrdU assay (*p<0.05, **P<0.01, Figure 2C). Moreover, metastatic and invasive abilities in HCC cells were investigated by transwell chamber assay. The results demonstrated that HCP5 knockdown in Hep3B and HCCLM3 cells resulted in alleviating the cell metastatic and invasive quantities (**p<0.01, ***P<0.001, Figure 2D, 2E), which indicated that the presence of HCP5 could functionally augment the metastatic and invasive abilities in HCC.

We showed that overexpression of HCP5 enabled the proliferation, metastatic and invasive ability of HCC, the exact modulatory process is still not clear. Therefore, flow cytometry was conducted to further determine whether a change in HCP5 expression could affect cell apoptosis. The flow cytometry results showed that the apoptosis rate was negatively proportional to the HCP5 expression level, which meant that down-regulation of HCP5 lead to a higher apoptosis level (***p<0.001, Figure 2F). Additionally, we estimated the expression level of EMT related proteins (E-cadherin and vimentin) by Western blot analysis. As shown in Figure 2G, knockdown of HCP5 resulted in high expression of E-cadherin and down-regulation of vimentin in HCC cell lines (Hep3B and HCCLM3), which illustrated that down-regulation of HCP5 might inhibit HCC cell metastasis and invasion via inactivating the EMT process.

**Figure 3:**
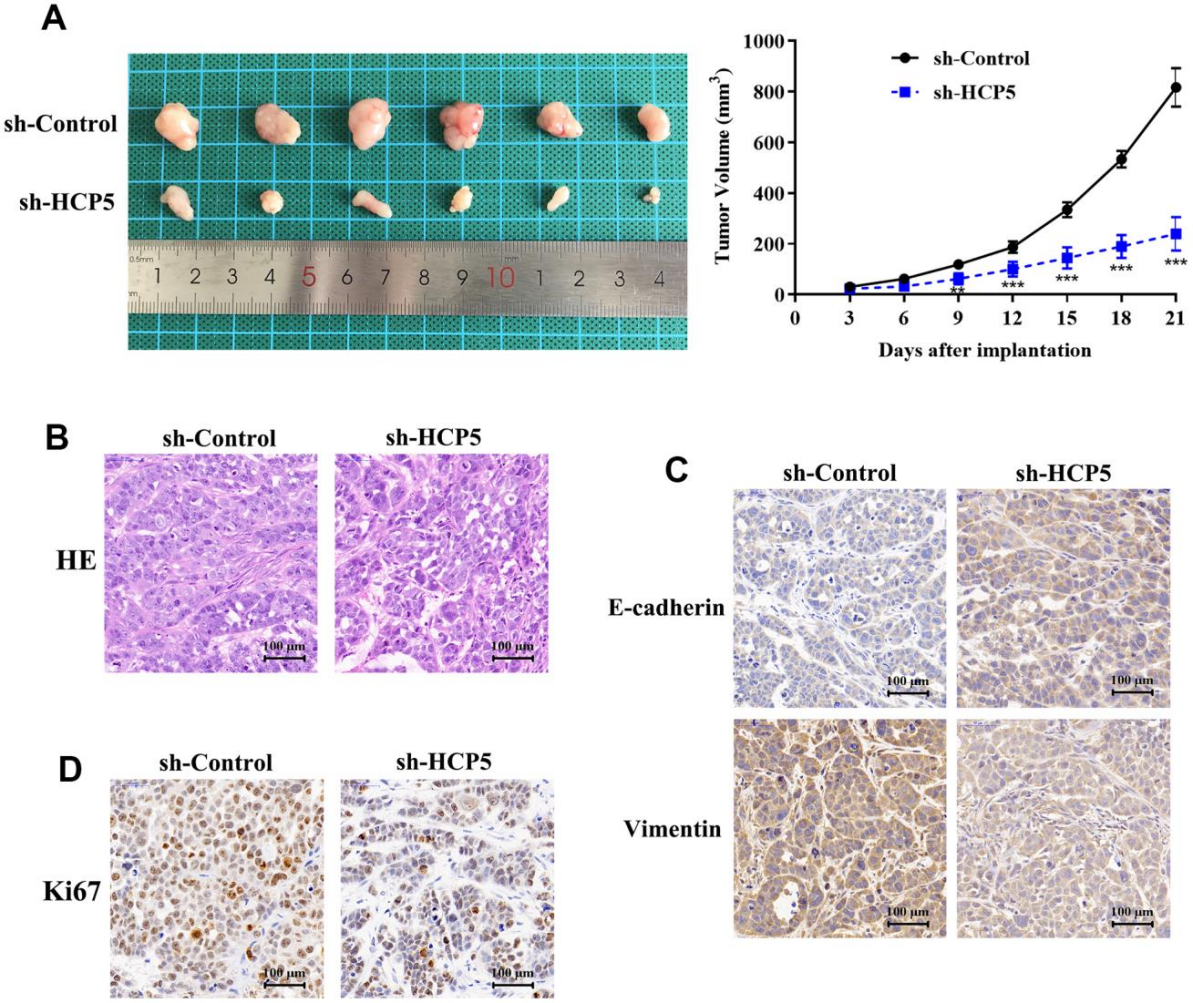
**LncRNA HCP5 promotes the growth and migration of HCC *in vivo*.** (**A**) Tumor growth curve suggesting that the knockdown of HCP5 results in lower tumor growth *in vivo* (**p<0.01, ***p<0.001). (**B**) Results of hematoxylin and eosin (HE) staining. (**C**) Immunohistochemistry demonstrating the expression of E-cadherin and vimentin between tissues transfected with sh-control and sh-HCP5. (**D**) Immunohistochemistry illustrating that the knockdown of HCP5 results in lower a proliferation rate in HCC tissue when compared to the control group.

Although we have found evidence that HCP5 could promote HCC proliferation, metastasis and invasion *in vitro*, the *in vivo* effect of HCP5 on HCC progression still needs to be elucidated. Consequently, we injected HCCLM3 cells into nude mice to establish an HCC animal model. The tumor volume was recorded every 3 days after establishing the xenograft. The tumor volume of the sh-HCP5 transfected group was lower than that of the control group (Figure 3A), which further indicated that down-regulation of HCP5 could slow down HCC progression. Next, tumors were excised from nude mice and sectioned. HE-staining was used to evaluate the differences between controls and mice with down-regulated levels of HCP5. Consistent with the *in vitro* findings, knockdown of HCP5 destroyed the HCC tumor tissues (Figure 3B). Additionally, the metastasis phenotype was also examined and the results shown in Figure 3C suggested that knockdown of HCP5 led to higher E-cadherin expression and lower vimentin expression when compared with the sh-NC transfected group (Figure 3C). Moreover, Ki67 was utilized to estimate the HCC proliferation *in vivo*. Consistent with our hypothesis, down-regulation of HCP5 significantly reduced the number of cells that were positive for Ki67 staining (Figure 3D), which demonstrated that HCP5 is a pivotal factor in HCC progression.

### MiR-29b-3p interacted with HCP5

**Figure 4:**
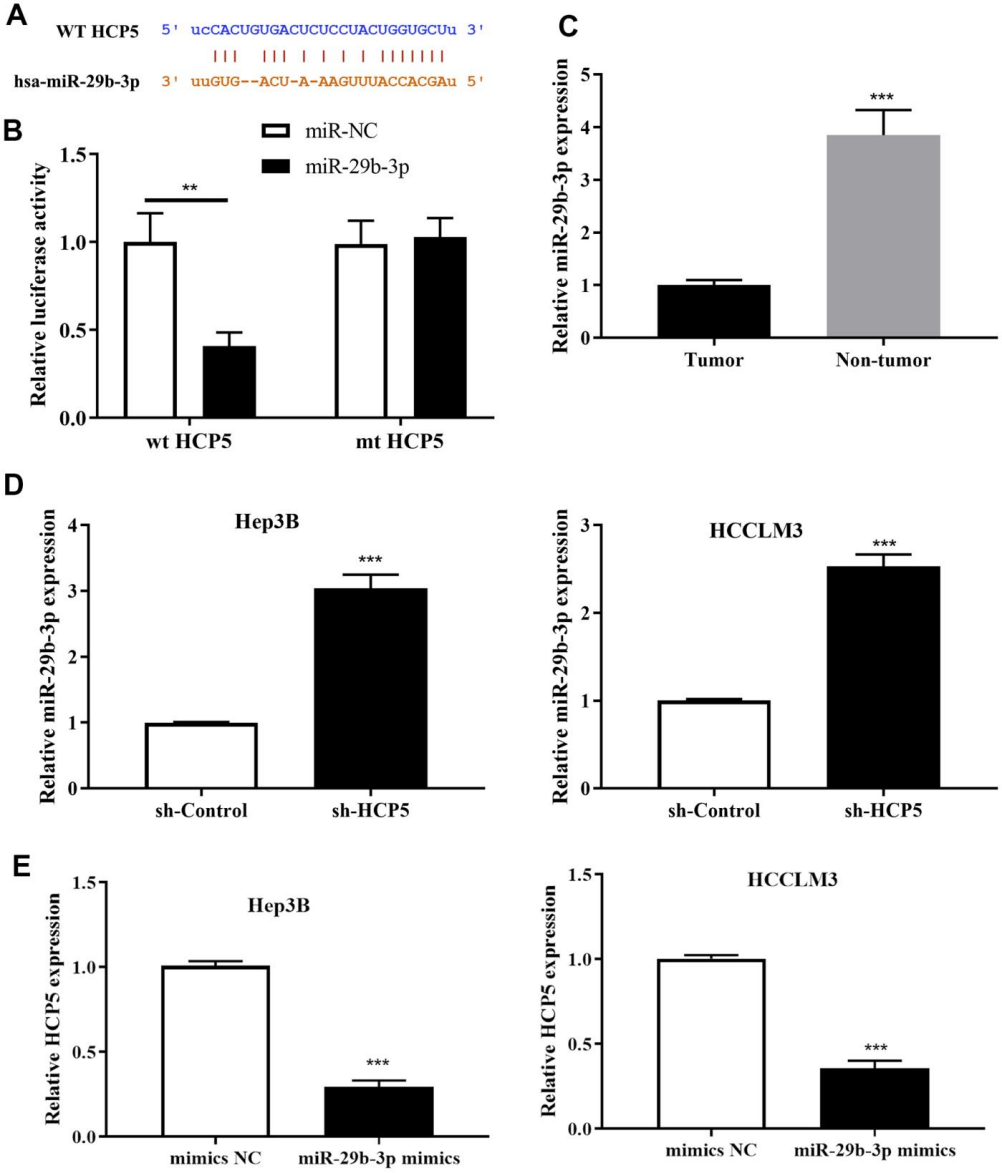
**MicroRNA miR-29b-3p is a negatively regulatory target gene of lncRNA HCP5.** (**A**) Bioinformatics predictions demonstrated that a direct HCP5 binding site existed in miR-29b-3p. (**B**) Dual luciferase assay demonstrating that miR-29b-3p and HCP5 directly interacted (**p<0.01). (**C**) MiR-29b-3p is expressed in lower-than-usual quantities in hepatocellular carcinoma (HCC) tumor tissues when compared to non-tumor tissues (***p<0.001, U6 served as the internal control). (**D**) Results of qRT-PCR showing that the knockdown of HCP5 increases the expression of miR-29b-3p both in HCCLM3 and Hep3B cell lines (***p<0.001). (**E**) Results of qRT-PCR showing that up-regulation of miR-29b-3p prohibits the expression of HCP5 (***p<0.001).

To further explore the downstream molecular regulatory mechanism of HCP5, we employed StarBase v3.0, miRanda and LncBase to identify a potential target gene of HCP5. The intersection of prediction targets of the three databases showed that there was a covalent binding fragment of HCP5 wild type binding sites on the miR-29b-3p gene sequence (Figure 4A), which encouraged us to further investigate the exact correlation between miR-29b-3p and HCP5 in HCC. The dual luciferase assay demonstrated a significant reduction in luciferase activity between wt-HCP5 and miR-29b-3p, which indicated that miR-29b-3p can be directly bound to HCP5 in HCC (**p<0.01, Figure 4B). The expression level of miR-29b-3p in HCC tissues and adjacent normal tissues was also determined. As expected, miR-29b-3p was expressed in low quantities in HCC tissues when compared to normal tissues (**p<0.001, Figure 4C, U6 served as the internal control), from which we speculated that miR-29b-3p might serve as a tumor suppressor in HCC. Also, the expression level between miR-29b-3p and HCP5 was analyzed through qRT-PCR and the results showed that miR-29b-3p and HCP5 were negatively correlated (Figure 4D, 4E). The results presented in Figure 4D show that knockdown of HCP5 increases miR-29b-3p expression (***p<0.001). While treated with miR-29b-3p mimics, HCP5 was found to be down-regulated (p<0.05, Figure 4E), which suggested that miR-29b-3p and HCP5 were in a negative feedback regulation cycle.

### MiR-29b-3p prevents HCC cell progression brought by HCP5

**Figure 5:**
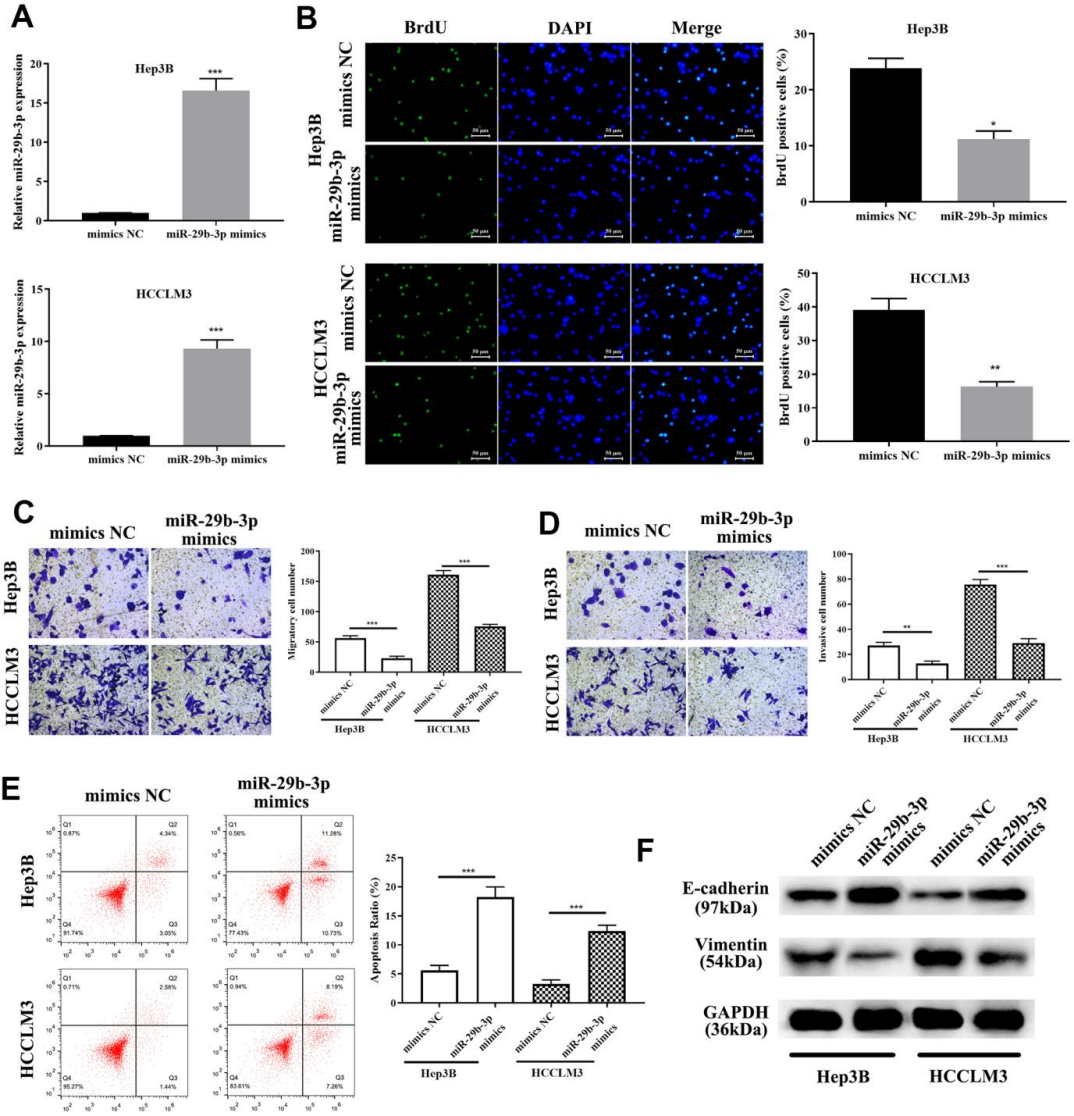
**miR-29b-3p precludes hepatocellular carcinoma cell proliferation and migration through enhancing apoptosis and inhibiting the epithelial-mesenchymal transition (EMT) progress.** (**A**) After treatment with miR-29b-3p mimics, HCCLM3 and Hep3B cell lines expressed high quantities of miR-29b-3p when compared to the mimics NC group. (**B**) Cells with high quantities of miR-29b-3p demonstrating a lower proliferation rate (*p<0.05, **p<0.01). (**C**, **D**) miR-29b-3p significantly reduces hepatocellular carcinoma (HCC) cell migration and invasion (**p<0.01, ***p<0.001). (**E**) Cell apoptosis is improved by miR-29b-3p (***p<0.001). (**F**) Western blot showing that overexpression of miR-29b-3p results in up-regulation of E-cadherin and reduced level of vimentin.

To explore the exact role of miR-29b-3p in HCC, miR-29b-3p mimics and mimics NC were used to treat HCC cells (HCCLM3 and Hep3B). QRT-PCR was conducted to evaluate the miR-29b-3p expression level after treatment of the above-mentioned reagents. MiR-29b-3p was highly expressed after treatment of HCC cells with miR-29b-3p mimics compared to the mimics NC group (***p<0.001, Figure 5A). BrdU assay demonstrated that highly expressed miR-29b-3p resulted in a lower proliferative rate (*p<0.05, **p<0.01 compared to mimics NC, Figure 5B), which directly illustrated that miR-29b-3p has the ability to prevent HCC cell proliferation. Additionally, the cell metastatic and invasive capacity were alleviated when HCC cell lines (HCCLM3 and Hep3B) were treated with miR-29b-3p mimics (**p<0.01, ***p<0.001, Figure 5C, 5D). Thus, these results collectively showed that miR-29b-3p can inhibit HCC cell proliferation, metastasis and invasion, thereby preventing the exacerbation of HCC. Next, flow cytometry was conducted to examine the effect of miR-29b-3p on cell apoptosis. Our data demonstrated that the apoptosis rate was higher in the miR-29b-3p mimics group (***p<0.001 compared with mimics NC, Figure 5E), which suggested that miR-29b-3p prevented cell growth through improving cell apoptosis. Furthermore, Western blot analysis demonstrated that high levels of miR-29b-3p led to up-regulation of E-cadherin and down-regulation of vimentin (Figure 5F).

**Figure 6:**
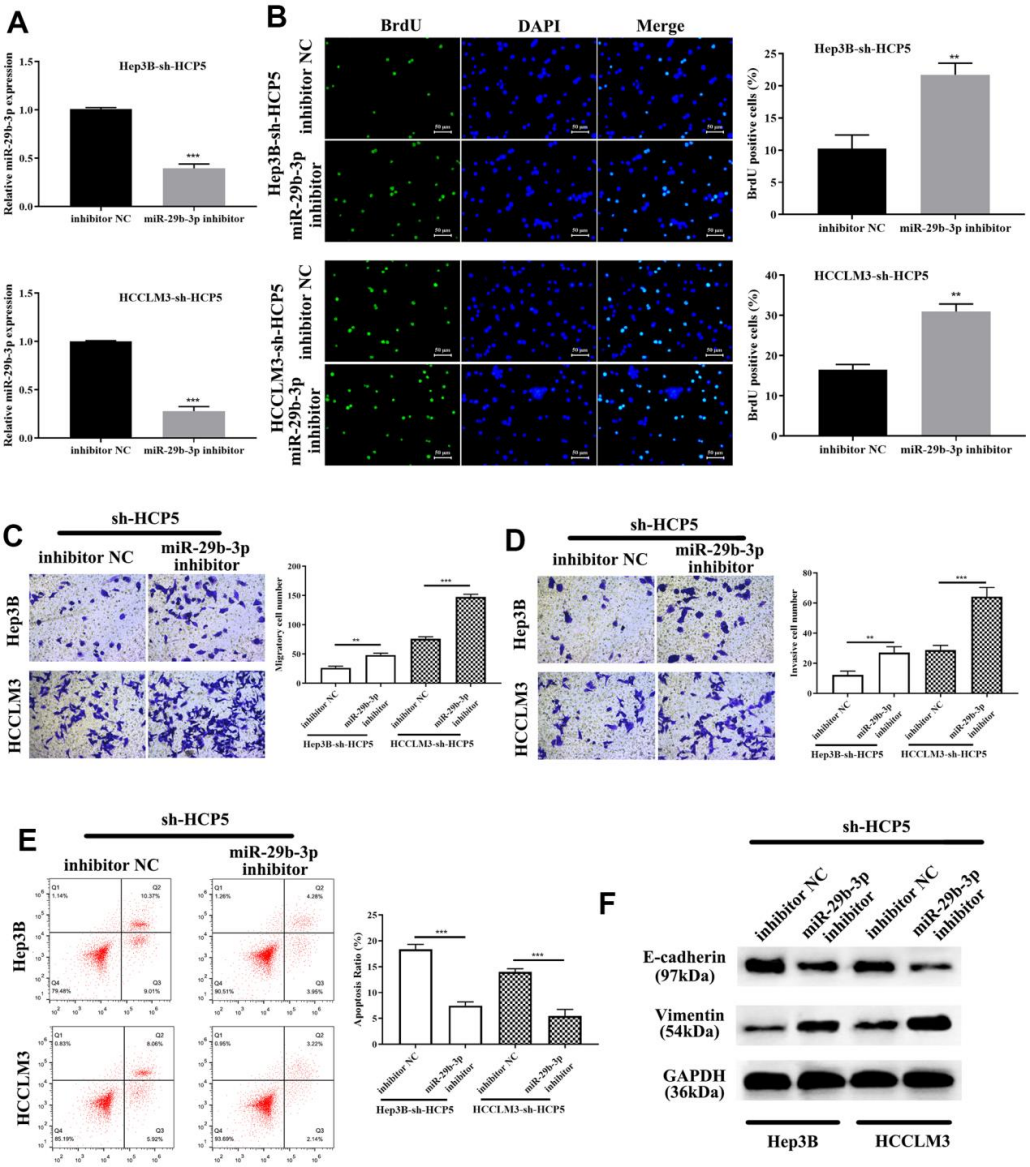
**miR-29b-3p reverses HCC proliferation, migration and invasion that is attenuated by sh-HCP5.** (**A**) The quantities of miR-29b-3p reduce in HCP5 knockdown cells when treated with an miR-29b-3p inhibitor. (**B**) miR-29b-3p is up-regulated when HCP5 is knocked down, and when cells were treated with miR-29b-3p inhibitor, the proliferation increased both in HCCLM3 and Hep3B cell lines (**p<0.01). (**C**, **D**) Down-regulation of miR-29b-3p significantly promotes cell migration and invasion (**p<0.01, ***p<0.001). (**E**) Cell apoptosis decreases after administration of miR-29b-3p inhibitor to two cell lines that were transfected with sh-HCP5. (**F**) miR-29b-3p inhibitor restores the EMT progress induced by sh-HCP5 both in HCCLM3 and Hep3B cell lines.

To investigate the correlation between miR-29b-3p and HCP5, miR-29b-3p inhibitor was given to sh-HCP5-transfected HCCLM3 cells. QRT-PCR showed that knockdown of HCP5 resulted in higher levels of miR-29b-3p, while, when treated with an miR-29b-3p inhibitor, the expression of miR-29b-3p decreased (***p<0.001, Figure 6A). In addition, the proliferation of miR-29b-3p inhibitor-treated HCCLM3 cells transfected with sh-HCP5 was determined using BrdU assay, and the result showed that when treated with an miR-29b-3p inhibitor, the proliferative rate significantly increased (**p<0.01, Figure 6B), which indicated that miR-29b-3p was a tumor suppressor of HCC. Moreover, the transwell assay demonstrated that the miR-29b-3p inhibitor further promoted the metastatic and invasive capacity attenuated by sh-HCP5 (**p<0.01, ***p<0.001, Figure 6C, 6D). Further studies of the mechanism were investigated through flow cytometry and Western blot analysis. The data showed that an miR-29b-3p inhibitor prevented cell apoptosis (***p<0.001, Figure 6E), which indicated that an miR-29b-3p inhibitor promoted cell growth through preventing apoptosis. Western blot analysis was conducted to determine the expression of EMT-correlated proteins (E-cadherin and vimentin), the results showed that when treated with an miR-29b-3p inhibitor, the expression of vimentin was increased while that of E-cadherin was decreased (Figure 6F). These data collectively showed that miR-29b-3p blocked cell growth, metastatic and invasive capacity via promoting apoptosis, up-regulating E-cadherin and down-regulated Vimentin.

### DNMT3A was proved as a target gene of miR-29b-3p

**Figure 7:**
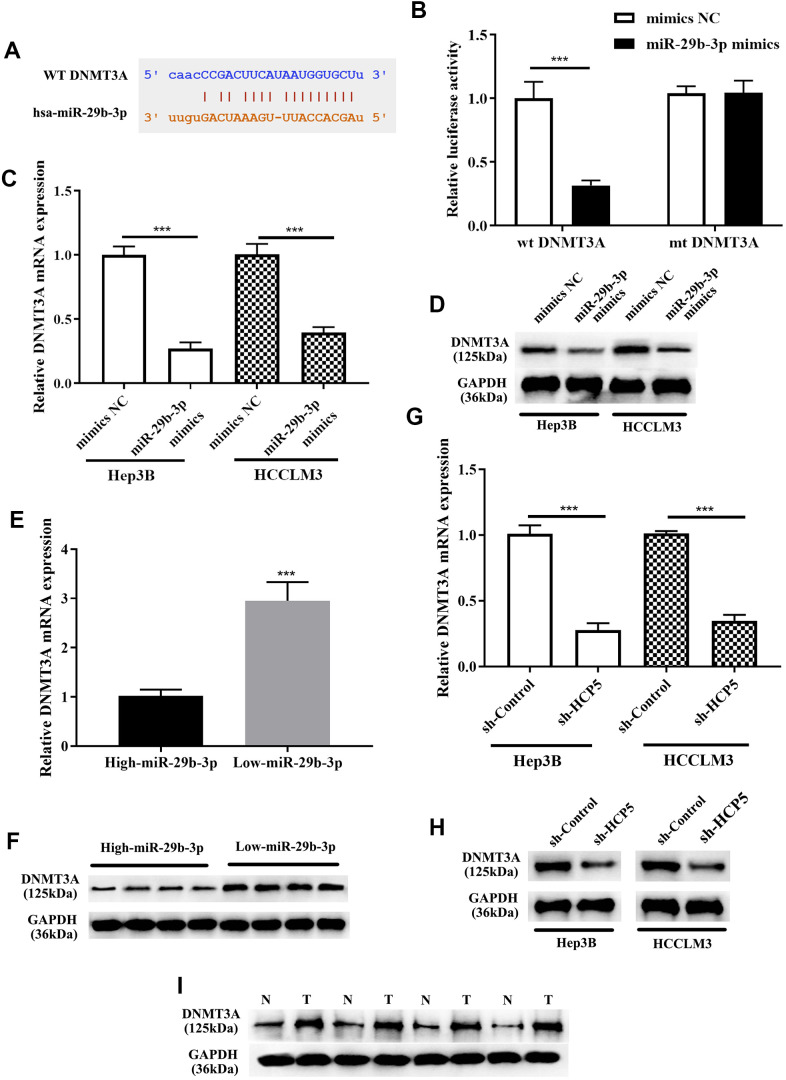
**DNMT3A is a negative regulatory target gene of miR-29b-3p, and DNMT3A promotes hepatocellular carcinoma proliferation, metastasis and invasion.** (**A**) Bioinformatics predictions showed that there is a direct binding site of miR-29b-3p on DNMT3A. (**B**) Dual luciferase assay demonstrating that there is a direct interaction between DNMT3A and miR-29b-3p (***p<0.001). (**C**, **D**) The results of qRT-PCR and Western blot analysis showing that overexpression of miR-29b-3p leads to down-regulation of DNMT3A in both HCCLM3 and Hep3B cells (***p<0.001). (**E**, **F**) qRT-PCR and Western blot analysis demonstrating that the expression of DNMT3A was negatively related to miR-29b-3p levels in HCC tissues. (**G**, **H**) The expression of DNMT3A was in proportion to that of HCP5. (**I**) Comparison of the expression of DNMT3A in HCC tissues (T) and non-tumor tissues (N) by Western blot analysis.

For further exploration of the mechanism by which miR-29b-3p modulates the progression of HCC, Targetscan, miRanda, and miRWalk databases were used to identify direct target genes of miR-29b-3p. Subsequently, DNMT3A was found as the downstream target of miR-29b-3p. As shown in Figure 7A, the bioinformatics prediction demonstrated that DNMT3A was indeed a potential target gene of miR-29b-3p. IN addition, the dual luciferase assay further showed that there was a direct interaction between miR-29b-3p and DNMT3A (***p<0.001, Figure 7B). Western blot and qRT-PCR analysis exerted that expression level of DNMT3A was reduced when treated miR-29b-3p mimics. The results showed that high levels of miR-29b-3p inhibited the expression of DNMT3A (***p<0.001, Figure 7C, 7D). Moreover, qRT-PCR and Western blot results demonstrated that tissues with down-regulated miR-29b-3p had higher expression levels of DNMT3A. High expression of miR-29b-3p resulted in lower expression of DNMT3A, which indirectly elucidated that the expression of DNMT3A was inverse correlated to miR-29b-3p. (Figure 7E, 7F). Furthermore, qRT-PCR and Western blot analysis showed that knockdown of HCP5 resulted in lower expression levels of DNMT3A at mRNA and protein levels when compared with sh-control both in Hep3B and HCCLM3 cells (Figure 7G, 7H). Next, expression levels of DNMT3A in HCC tissues were determined through Western blot analysis, and the data demonstrated that DNMT3A was up regulated in HCC tissues when compared to adjacent normal tissues (Figure 7I). The results mentioned above collectively illustrated that DNMT3A is a crucial participating factor in modulating HCC progression.

### Overexpression of DNMT3A promotes HCC cell progression that weakened by miR-29b-3p or sh-HCP5

**Figure 8:**
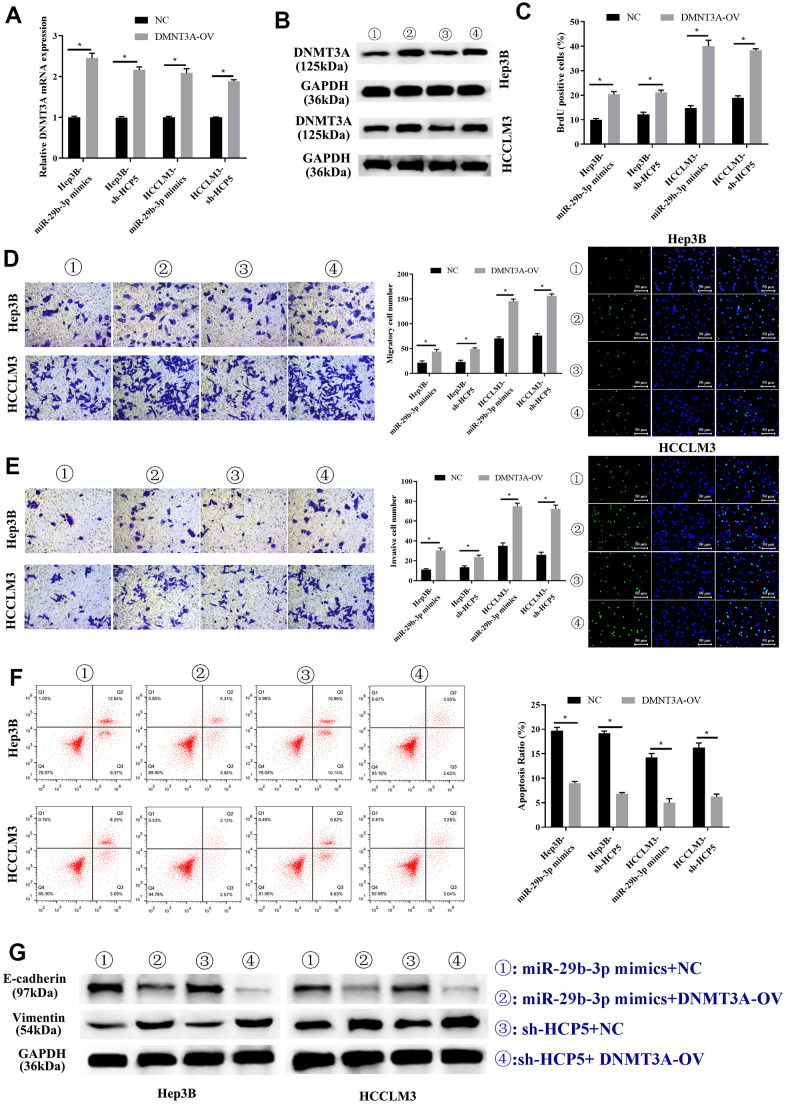
**Overexpression of DNMT3A promotes hepatocellular carcinoma cell progression weakened by miR-29b-3p or sh-HCP5.** (**A**) Hep3B and HCCLM3 cells with up-regulation of miR-29b-3p or knockdown of HCP5 and that were transfected with NC or DNMT3A up-regulated plasmid (DNMT3A-OV) were evaluated by qRT-PCR (*p<0.05). (**B**) The expression of DNMT3A in Hep3B and HCCLM3 cell lines transfected with NC or DNMT3A-OV examined by Western blot analysis for miR-29b-3p overexpression or HCP5 knockdown (*p<0.05). (**C**) The cell proliferation rate increases when treated with DNMT3A-OV (*p<0.05). (**D**, **E**) DNMT3A promotes the cell metastatic and invasive capacity (*p<0.05). (**F**) DNMT3A promotes cell proliferation through inhibiting cell apoptosis (*p<0.05). (**G**) DNMT3A promotes epithelial-mesenchymal transition (EMT) progress by increasing E-cadherin and decreasing vimentin.

Previous studies have shown that DNMT3A played a role in HCC, therefore, we proposed that up-regulation of DNMT3A might alter the HCC progression inhibited by miR-29b-3p. Consequently, DNMT3A-OV and NC were transfected into HCC cell lines (HCCLM3 and Hep3B) to up-regulate the expression of DNMT3A. In addition, miR-29b-3p mimics and sh-HCP5 transfection were used to determine the correlation of miR-29b-3p, HCP5, and DNMT3A. As shown in Figure 8A, qRT-PCR demonstrated that the DNMT3A expression decreased when miR-29b-3p was overexpressed, when co-given overexpression plasmid of DNMT3A, the expression level significantly increased both in Hep3B and HCCLM3 cell lines (*p<0.05). Furthermore, in Hep3B and HCCLM3 cells, knockdown of HCP5 by sh-HCP5 presented decreased level of DNMT3A when merely transfected with NC, while the expression level increased when given DNMT3A up-regulation plasmid transfection (*p<0.05). Consistent with these results, western blot analysis showed that the expression of DNMT3A was down-regulated in miR-29b-3p overexpression or HCP5 knockdown cells, and was up-regulated when transfected with a DNMT3A overexpression plasmid (Figure 8B). Subsequently, the BrdU assay was used to determine the effect of DNMT3A on HCC proliferation, and the results showed that overexpression of DNMT3A led to increased cell growth (*p<0.05, Figure 8C). Next, the cell metastatic and invasive ability were examined through the transwell chamber assay, and the data suggested that both the metastatic and invasive capacity were inhibited, both in Hep3B and HCCLM3 cells, when transfected NC in the miR-29b-3p overexpression group and HCP5 knockdown group, while the migration and invasion significantly improved when cells were transfected with an up-regulation plasmid of DNMT3A with overexpression of miR-29b-3p or down-regulation of HCP5 (*p<0.05, Figure 8D, 8E). Further exploration of the effect of DNMT3A on regulating proliferation, invasion and migration was performed by flow cytometry and examination of the expression level of EMT-correlated proteins (E-cadherin and vimentin). Flow cytometry showed that DNMT3A promoted cell growth through prohibiting cell apoptosis (*p<0.05, Figure 8F). Simultaneously, Western blot analysis showed that high regulation of DNMT3A could up-regulate vimentin and decrease the expression of E-cadherin (Figure 8G), which suggested that DNMT3A enhanced cell migration and invasion via stimulating the EMT process. These data demonstrated that the proliferation, metastatic and invasive capacity of HCC was augmented when DNMT3A was highly expressed, the mechanism of which involved the prevention of apoptosis and acceleration of the EMT process.

### HCP5/miR-29b-3p/DNMT3A axis augments HCC progression via activating AKT phosphorylation

**Figure 9:**
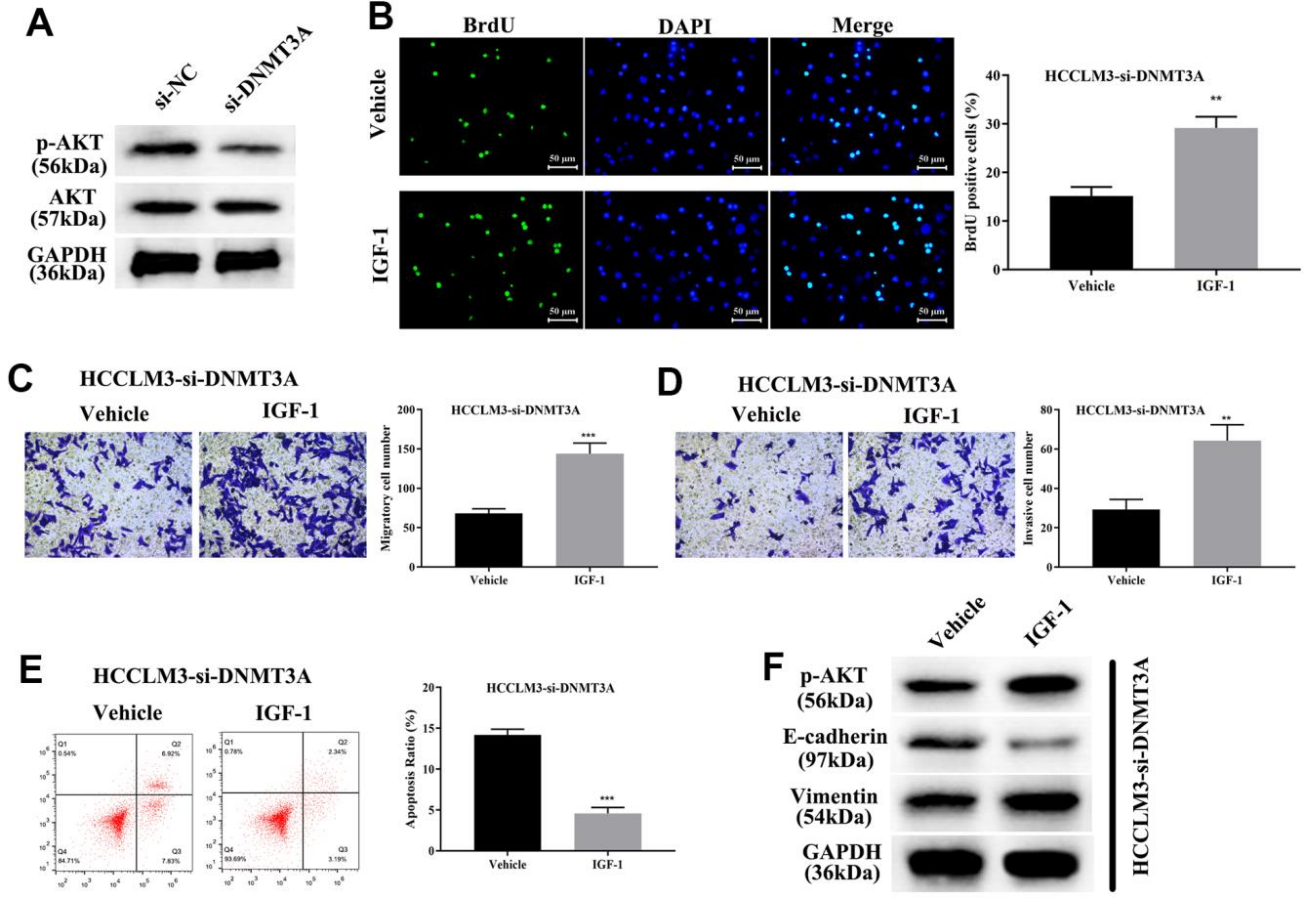
**HCP5/miR-29b-3p/DNMT3A axis augments hepatocellular carcinoma progression by activating AKT phosphorylation.** (**A**) Knockdown of DNMT3A decreases the phosphorylation of AKT. (**B**) Cell proliferation was augmented when treated with AKT activator IGF-1 (**p<0.01). (**C**, **D**) IGF-1 reverses cell migration and invasion that was decreased by down-regulation of DNMT3A (**p<0.01, ***p<0.001). (**E**, **F**) IGF-1 prevents cell apoptosis and activates the epithelial-mesenchymal transition (EMT) progress in HCCLM3-si-DNMT3A cells (***p<0.001).

Current studies have shown that DNMT3A can methylate the promoter region of the PTEN gene, thereby resulting in inhibition of transcription of the PTEN gene and decreased expression of PTEN protein, finally elevating the phosphorylation level of AKT [27]. Thus, DNMT3A plays a pivotal role in modulating tumorigenesis through activating the PI3K/AKT pathway in liver cancer and lung cancer [27, 32]. To identify the modulatory function of AKT phosphorylation in HCP5/miR-29b-3p/DNMT3A, we first confirmed that knockdown of DNMT3A significantly decreased AKT phosphorylation by Western blot analysis (Figure 9A). Our data showed that the phosphorylation level of AKT was proportional to DNMT3A. Next, IGF-1, an AKT activator was used to treat HCC cell lines with si-DNMT3A transfection, cell proliferation was determined through BrdU assay and the results demonstrated that activation of AKT enhanced cell growth that was weakened by si-DNMT3A (**p<0.01, Figure 9B). A transwell chamber assay was used to determine the effect of AKT on regulating the HCC metastatic and invasive capacity. The data demonstrated that AKT activation augmented the metastatic and invasive capacity of HCC cells that were attenuated by si-DNMT3A (***p<0.001, **p<0.01, Figure 9C, 9D). Further investigation showed that activation of AKT phosphorylation led to a reduction in cell apoptosis that strengthened the EMT process that was impaired by si-DNMT3A (***p<0.001, Figure 9E, 9F), which proved that the HCP5/miR-29b-3p/DNMT3A axis played a role in AKT phosphorylation in regulating HCC progression.

## DISCUSSION

Accumulated incidences of HCC have roused attention and increased investigation of HCC has been performed to search for new curatives. Due to the occult onset of HCC and the lack of specificity of early symptoms, the vast majority of patients have entered the advanced stage or developed metastasis when diagnosed. Therefore, finding early diagnostic biomarkers of HCC and understanding the underlying biological mechanism is of great significant in the HCC therapeutic area. Recent studies have revealed the modulatory role of non-coding RNAs (long non-coding RNAs and microRNAs) in various types of carcinomas [33–36].

Our findings also made effect to discover novel diagnostic biomarkers for HCC, which might provide novel insight in HCC diagnosis. Using the StarBase database, data from the TCGA was analyzed, suggesting that HCP5 was significantly and highly expressed in HCC tissues compared to normal tissues. Furthermore, previous studies also suggested that HCP5 had a pathological association with HCV-related HCC [19]. Yet, only few studies have investigated HCP5 in HCC. Therefore, we postulated that HCP5 probably had a specific role in mediating the course of HCC. In this study, high expression of HCP5 was detected both in HCC tissues and cell lines. To further study whether HCP5 was associated with HCC in humans, we analyzed the correlation of HCP5 expression and some clinical parameters. According to our collected human HCC data, we discovered that the expression of HCP5 was closed related with HCC tumor size, vascular invasion, and the TNM stage (Table 1), which further suggested the possibility that high levels of HCP5 did have an accelerating effect in mediating the course of HCC. Furthermore, we elucidated that HCP5 promoted HCC progression (proliferation, metastasis and invasion) via inhibiting apoptosis and activating the EMT process both *in vitro* and *in vivo*, which further proved our assumption that HCP5 served as a promoting factor in HCC.

Previous studies reported that lncRNAs could serve as ceRNAs for miRNAs and thereby modulate tumor progression [37–39]. Deeper studies of the downstream molecular regulatory targets of HCP5 deserve further studying. Regarding the in-depth investigation of HCP5, we found that miR-29b-3p was a target gene of HCP5 via bioinformatics prediction and a dual-luciferase assay. Also, miR-29b-3p was found as a tumor suppressor and negatively regulatory factor of HCP5 in HCC. Additionally, Brdu assay, transwell assay, qRT-PCR, Western blot analysis, and flow cytometry collectively illustrated that HCP5 promoted HCC progression via repressing miR-29b-3p. Thus, we established a modulatory axis of HCP5/miR-29b-3p in HCC, which requires further studies.

DNA methyltransferase 3A (DNMT3A) has been recognized as a promoting factor in various cancer. However, whether DNMT3A has a pathological role in HCC still needs further studies. In this study, we identified for the first time that DNMT3A participated in promoting the progression of HCC. Using Targetscan, miRanda and miRWalk, we found that DNMT3A was a direct target gene of miR-29b-3p, and a dual luciferase assay further confirmed this finding. Furthermore, DNMT3A inversely associated with the expression of miR-29b-3p in HCC cells. Down-regulation of DNMT3A reversed the biological effects of HCP5 and miR-29b-3p on HCC. Many studies have shown that AKT phosphorylation is regulated by DNMT3A via modulating the PTEN gene in various carcinomas, including pancreatic cancer and lung cancer [40–42]. These findings encouraged us to further investigate whether DNMT3A also exerted its effect by mediating AKT. Western blot analysis showed that DNMT3A also regulated the activation of AKT in HCC, which further confirmed and optimized the modulatory axis in HCC as HCP5/miR-29b-3p/DNMT3A/AKT.

Conclusively, our findings suggested that HCP5 was overexpressed in HCC, and accelerated cell proliferation, metastatic and invasive capacity via preventing apoptosis and promoting the EMT process through the HCP5/iR-29b-3p /DNMT3A/AKT axis. Therefore, by targeting HCP5 inhibition, we could suppress the progression of HCC. This discovery might provide novel strategies for early diagnosis and better prognostic methods for HCC.

## MATERIALS AND METHODS

### Patients’ samples and cell culture

HCC tissues and adjacent normal tissues were collected through surgical resection from HCC patients in the Wuxi Second Hospital (Wuxi, China) and The Affiliated BenQ Hospital of Nanjing Medical University (Nanjing, China). Extracted tissues were immediately frozen in liquid nitrogen. All experiments related to human participants were conducted in agreement with the ethical standards of the ethics committee of Wuxi Second Hospital (Wuxi, China) and The Affiliated BenQ Hospital of Nanjing Medical University (Nanjing, China) as well as the 1964 Helsinki Declaration. All patients involved signed the informed consent documents. Subsequently, clinicopathological parameters and demographic data from patients were collected and presented in Table 1. Human hepatic cell line (LO2) and human HCC cell lines (Hep3B, HCCLM3, Huh7, MHCC-97H, and HepG2) were cultured in Dulbecco’s Modified Eagle’s Medium (DMEM) with 10% FBS at 37° C and 5% CO_2_.

### Quantitative reverse transcriptase polymerase chain reaction (qRT-PCR)

HCC tissues and cell lines (Hep3B, HCCLM3) that received different treatments were collected in the logarithmic growth phase and mixed with 1 mL of TRIzol. Chloroform was added for 15-min at room temperature. Then, isopropyl alcohol was added and the solution was centrifuged to obtain the RNA sediment. Finally, the RNA extraction was dried and stored at -80° C till experiments. QRT-PCR analyses of HCP5, miR-29b-3p and DNMT3A were performed using the PrimeScript RT reagent Kit and SYBR Prime Script RT-PCR Kits based of the manufacturer’s guidelines. The transcription level was subsequently analyzed by the 2−ΔΔCt method. GAPDH was used as the internal control of HCP5, DNMT3A, and U6 was used as the internal control of miR-29b-3p. The detailed primer sequences are listed in Table 2. All assays were performed in triplicate.

**Table 2:** The nucleotides in this study.

**Name**	**Sequences**
HCP5 forward primer	5’-TGAGAGCAGGACAGGAAAA-3’
HCP5 reverse primer	5’-CCAACCAGACCCTAAGTGA-3’
miR-29b-3p forward primer	5’-AGGCTAGCACCATTTGAAATC-3’
miR-29b-3p reverse primer	5’-GAGAGGAGAGGAAGAGGGAA-3’
DNMT3A forward primer	5’-TGACACGCCAAAGGACCCTG-3’
DNMT3A reverse primer	5’-GCTCACTCCGCTTCTCCAAGT-3’
U6 forward primer	5’-CTCGCTTCGGCAGCACA-3’
U6 reverse primer	5’-GGATGGTGATGGTTTGGTAG-3’
GAPDH forward primer	5’-ATGGGGAAGGTGAAGGTCGG-3’
GAPDH reverse primer	5’-GACGGTGCCATGGAATTTGC-3’
si-HCP5	5’-GCTGGTCTCTGGACACATACTCTCGAGA GTATGTGTCCAGAGACCAGCTTTTTG-3’
miR-29b-3p mimics	5’-UAGCACCAUUUGAAAUCAGUGUU-3’
mimics NC	5’-UUCUCCGAACGUGUCACGUTT-3’
miR-29b-3p inhibitor	5’-AACACUGAUUUCAAAUGGUGCUA-3’
Inhibitor NC	5’-CAGUACUUUUGUGUAGUACAA-3’
si-DNMT3A	5’-GCCTCAGAGCTATTACCCAATCTCGAGAT TGGGTAATAGCTCTGAGGCTTTTTG-3’

### Fluorescence *in situ* hybridization (FISH)

The FISH experiment was conducted using the lncRNA FISH kit (Ruibo, Guangzhou, China) based on the manufacturer's guidelines. After fixation with 4% formaldehyde for 30 min, Hep3B cells were treated with the buffers for permeabilization that were included in the kit. Then, cells were mixed with a hybridization buffer by using 40 nmol/L of the FISH probe of lncRNA HCP5, and incubated for 2 min at 37° C. After washing with 2x SSC buffer, the cells were counterstained using Prolong Gold Antifade Reagent with DAPI to stain the nuclei. Finally, cells were visualized using the Zeiss Confocal Microscope Imaging System (Carl Zeiss, Germany).

### Western blot analysis

Cells were collected and lysed in lysis buffer on ice for 30 min. Then, the protein content was determined using the BCA Kit. Proteins were then mixed with loading buffer and boiled for 5 min. Subsequently, proteins were separated by SDS-PAGE and transferred onto polyvinyl difluoride membranes (Bio-Rad Laboratories, USA) after separated. Membranes were incubated with antibodies directed against DNMT3A, AKT, p-AKT, E-cadherin, vimentin and GAPDH, purchased from Abcam. GAPDH was used as an internal control. Proteins were visualized by an enhanced chemiluminescence reagent (Thermo Scientific, Waltham, MA, USA).

### Transfection of cells

Vectors encoding short-hairpin HCP5 (sh-HCP5) and the negative control (sh-control) were designed and synthesized by Geneseed Biotech (Guangzhou, China). Specific small RNAs targeting DNMT3A (si-DNMT3A) and small RNA negative control (si-NC), were purchased from Sangon Biotech Co., Ltd. (Shanghai, China). Sh-HCP5, sh-Control, si-DNMT3A, and si-NC were transfected into Hep3B and HCCLM3 cells to knockdown HCP5 and DNMT3A based on the manufacturer’s guidelines, respectively. MiR-29b-3p mimics and negative control (mimics NC) were obtained from Guangzhou RiboBio Co., Ltd. (Guangzhou, China) to transfect HCC cells according to the manufacturer’s guidelines. The DNMT3A open reading frame (GenBank: NM_001320892.2) was subcloned into the pcDNA3.1 expression vector (Invitrogen) to overexpress DNMT3A (DNMT3A-OV). All transfections were conducted using Lipofectamine2000 reagent (Invitrogen). The detailed siRNA sequences are listed in Table 2.

.

### Cell counting Kit-8 (CCK-8) assay

HCC cells were seeded into 96-well plates at a density of 1000 cells / per well. A total of 10 μL of CCK-8 solution (Dojindo, Tokyo, Honshu, Japan) was added to each well at 0h, 24h, 48h, and 72h. Finally, the absorbance at 450 nm was read using a microplate reader (Thermo-Fisher Scientific) after a 2 h incubation. All assays were performed in triplicate.

### Dual-luciferase assay

pGL3 vector (Promega Corporation, Madison, WI, USA) and the synthetic HCP5 and DNMT3A containing wild-type (WT) or mutated (Mut) region (Sangon, Shanghai, China) were utilized to construct the reporter plasmids, which were then co-transfected into cells with miR-29b-3p mimics using Lipofectamine 2000 following the manufacturer’s guidelines. In addition, a negative control was used to generate the control group. A Dual-Luciferase Reporter Assay System and luminometer were utilized to estimate the Renilla and Firefly luciferase activities after 48 h. All assays were performed in triplicate.

### Transwell chamber assay

For lucubration of the capacity of cell migration, 2 × 10^4^ cells were collected and transferred to the upper compartment of a Transwell chamber with an 8-μm pore size and a 24-well insert. In the upper chamber of each well, 250 μL of serum-free medium, containing 10 g/L bovine serum albumin was added. The lower chambers were loaded with 10% FBS. The cell migration capacity was assessed by the number of cells that migrated to the lower chamber. For invasion assays, the upper chamber was coated with Matrigel (BD Biosciences, San Jose, CA, USA). The other procedures were performed as in the migration assays. All assays were performed in triplicate.

### Apoptosis Level evaluated by flow cytometry

Flow cytometry was performed to study the effects on cell cycle and apoptosis after transfection. The Annexin V-FITC Apoptosis Detection Kit and Cell Cycle Detection Kit (Beyotime, Shanghai, China) were used to stain the cells. Cells were treated based on the manufacturer’s guidelines. All assays were performed in triplicate.

### Cell proliferation determination

After transfection, cells in the logarithmic growth phase were seeded into 24-well plates at 1x10^5^ cells per well. Subsequently, BrdU reagent was diluted using DMEM medium and added to stain cells according to manufacturer’s guidelines. Gray level images were acquired under a laser scanning microscope (Axioskop 2 plus, Carl Zeiss Co. Ltd., Jena, Germany). All assays were performed in triplicate.

### Animal studies

To further investigate the role of HCP5 on BALB/c nude mice with HCC, mice were raised and maintained in a pathogen-free environment at the Nanjing Medical University (Nanjing, China). All animal experiments were carried out in accordance with the National Institutes of Health guide for the care and use of laboratory animals, and approved by the ethics committee of Nanjing Medical University (Nanjing, China). Nude mice were then randomly allocated into two groups. One group was sh-Control group, and another was the sh-HCP5 group. A total of 100 μl of HCCLM3 cells (with sh-control or sh-HCP5 transfection) (1×10^6^ cells) in PBS was injected into the flanks of nude mice to establish a stable HCC animal model. Three weeks after injection, nude mice were euthanized, and HCC tumors were extracted for observation. Next, the tumor volume of each mice was recorded and tumors were photographed. Extracted HCC tissues were sectioned for immunohistochemical analysis.

### Immunohistochemical staining (IHC)

Sections were dewaxed, dehydrated, and rehydrated. Sections were incubated with the primary antibody (1:100, cell signal, Danvers, MA, USA) overnight at 4° C. Then, the biotinylated secondary antibody (Goldenbridge, Zhongshan, China) was applied according to the SP-IHC test.

### Statistical analyses

Data were expressed as the mean plus standard deviation (SD). Experiments were performed in triplicate. Statistically significant differences in different groups were evaluated by Student’s t test through SPSS (13.0) or Graphpad Prism 7.0. The Pearson correlation-based chi-squared test or Fisher’s exact test was utilized for analysis between the clinicopathologic characteristics and HCP5 expression. A two-tailed P < 0.05 indicated statistically significance.

### Ethics approval and consent to participate

All procedures performed in studies involving human participants were in accordance with the ethical standards of the Research Ethics Committee of Wuxi Second Hospital and The Affiliated BenQ Hospital of Nanjing Medical University and with the 1964 Helsinki declaration and its later amendments. All written informed consent to participate in the study was obtained from HCC patients for samples to be collected from them.
